# PD-L1 immunostaining scoring for non-small cell lung cancer based on immunosurveillance parameters

**DOI:** 10.1371/journal.pone.0196464

**Published:** 2018-06-06

**Authors:** Manuel A. Silva, Karen A. Ryall, Claudia Wilm, Jenifer Caldara, Hans Juergen Grote, Janet C. Patterson-Kane

**Affiliations:** 1 Department of Clinical Biomarkers and Companion Diagnostics, Merck KGaA, Darmstadt, Hessen, Germany; 2 Department of Pathology, Flagship Biosciences, Inc., Westminster, Colorado, United States of America; Catalan Institute of Oncology, SPAIN

## Abstract

Non-Small Cell Lung Cancer (NSCLC) is the leading cause of cancer death globally, and new immunotherapies developed and under development targeting PD-1/PD-L1 checkpoint inhibition require accurate patient selection to assure good clinical outcome. PD-L1 immunohistochemistry is the current biomarker assay used for patient selection, but still imprecise in predicting therapy response. Exploring this issue, we performed computational tissue analysis of PD-L1 immunostaining in procured NSCLC tissues (n = 50) using the Merck KGaA anti-PD-L1 clone MKP1A07310. Staining patterns and PD-L1 cut-off points were interrogated using relevant cancer immune-surveillance biomarkers. Groups with high PD-L1 expression levels (above 25/50% staining cut-off points) were enriched for a biomarker profile in the tumor-nest and microenvironment indicating escape from host-immunity, as represented by increased numbers of cells positive for CD8 and Granzyme B (immune-effectors), FOXP3 (immune-suppressive), and CD68 (*P* < 0.05). Manual analysis of PD-L1 staining patterns identified tumors with an immune-induced *reactive pattern* relevant for immunotherapy that would ordinarily be excluded by the arbitrary 25% staining threshold (*P* < 0.05). Conversely, some cases with completely or predominantly immune-independent *constitutive* PD-L1 staining patterns that indicate insensitivity to immunotherapy may have been incorrectly selected using this staining cut-off point criterion. Therefore, we propose differentiation of *reactive vs constitutive* PD-L1 staining patterns to improve the accuracy of this biomarker assay in selecting NSCLC patients for PD-1/PD-L1 immunotherapy.

## Introduction

Lung cancer is the most lethal cancer type worldwide, with non-small cell lung cancer (NSCLC) accounting for the majority (~85%) of deaths [1]. Late diagnoses of NSCLC contribute to the high mortality and poor prognoses for those with advanced disease, treated with platinum-based chemotherapy [2]. Novel immunotherapies that target the Programed Cell Death (PD)-1 /ligand (PD-L1) check-point have been approved for NSCLC based on improved overall response rates in clinical trials using therapeutic monoclonal antibodies (mAb) directed against PD-1 (Nivolumab and Pembrolizumab) and PD-L1 (Atezolizumab), while other anti-PD-L1 mAbs (Avelumab and Durvalumab) are at advanced stages of development [3–5]. With the current range of options, it is important to understand which patients will benefit from each new immunotherapy drug. PD-L1 immunohistochemistry (IHC) assays are the most advanced biomarker strategies in-place for patient screening, as elevated tissue expression of PD-L1 has been correlated with a higher chance of prolonged post-therapy survival [3, 4, 6]. However, this approach has attracted some skepticism given that significant number of patients, even in highly selected cohorts, have treatment outcomes that contradict predictions based on PD-L1 status [6]. The use of multiple antibodies and staining thresholds (1–50%) complicates PD-L1 IHC data comparisons [4, 6, 7]. Furthermore, we lack a standard scoring algorithm for PD-L1 expression patterns in tumor-nests and the tumor microenvironment (TME) [3, 8]. Finally, the biological relevance of PD-L1 staining cut-off points, including their relationship to other immune biomarkers, remains unknown.

The PD-L1 staining profile targeted by PD-L1/PD-1 immunotherapies should be a reactive pattern induced by interferon (IFN)-γ secreted by tumor infiltrating lymphocytes (TIL) at tumor-nest-TME interfaces [6, 8–10]. This reactive expression, from immune and tumor cells responsive to IFN-γ, differs from constitutive expression arising from cell-intrinsic genetic/epigenetic events. A strong and homogeneous constitutive PD-L1 tumor cell staining pattern in the absence of any appreciable TIL component would predict a futile PD-L1/PD-1 therapeutic intervention [6, 8]. Conversely, a reactive patchy PD-L1 pattern surrounded by T-cell rich areas implies restraint of a potentially anti-tumoral cytotoxic adaptive response, and a greater likelihood of anti-PD-L1/PD-1 therapeutic success [11, 12]. PD-L1 negative tumors without TIL will not respond to PD-1/L1 therapy unless cancer antigens are exposed for a T-cell response, while genetic events leading to inability to express PD-L1 in the presence of TIL should identify acquired resistance to PD-1/L1 therapy. Currently, none of the applied PD-L1 scoring algorithms account for patterns that indicate cancer escape from immune surveillance [6, 9].

This is the first publication to report IHC data for tumor-nest and TME compartments in NSCLC samples using the Merck KGaA anti-PD-L1 clone MKP1A07310 for Avelumab development together with an immune panel of effector and regulatory TIL [13], as well as to show the effects of PD-L1 staining cut-off points, and the stratification of biologically relevant PD-L1 staining patterns in tumor-nest and TME compartments. Using Computational Tissue Analysis (cTA^™^) capable of scoring entire sections, complementary to another NSCLC digital study [13], we expanded PD-L1 analyses to include all positively-stained TME cell types as all may support an immuno-suppressive environment. We demonstrated that simplistic thresholds based on PD-L1 staining amount over a whole section can exclude patients with lower amounts but potentially therapy-permissive patterns, while including cases with strong labeling that is not necessarily related to a restrained adaptive immune response.

## Materials and methods

### Tissues and immunohistochemistry

Formalin-fixed paraffin-embedded tissue blocks prepared using surgical resections from 50 treatment-naive NSCLC patients (Table 1) were procured from Indivumed GmbH (Hamburg, Germany) and processed in our Merck KGaA histopathology laboratory for IHC. Four-micrometer-thick serial sections were generated per tissue block and immunostained for CD8 (cytotoxic T-cells), FOXP3 (regulatory T-cells), CD56 (natural killer (NK) cells), Granzyme B (functionally active cytotoxic NK/T-cells), or CD68 (pan macrophage marker), with corresponding matching isotype controls. Staining was performed in a BenchMark XT immunostainer (Roche Diagnostics, Germany). Antigen retrieval pretreatments, primary antibody (Ab) working conditions, as well as detection and hematoxylin counterstaining methods are listed in Table 2. Tris-EDTA buffer pH 8 for mild and standard CC1 (Cat. 950–124), UltraView Universal DAB (Cat. 760–500), the OptiView DAB IHC Detection kit (Cat. 760–700), and Hematoxylin II (Cat. 790–2208) were purchased from Ventana Medical Systems (Roche Diagnostics, Germany) and used according to the manufacturer’s instructions. Additional serial sections were immunostained under the conditions presented in Table 2 for PD-L1 (clone MKP1A07310), with respective matching isotype controls. Staining was performed in a Dako AutostainerLink 48 instrument (Hamburg, Germany). EnVision FLEX/HRP DAB detection kit (Cat. SM802), TRS high pH (Cat. K8004), and Hematoxylin EnVision FLEX (Cat. SM806) were purchased from Dako (Hamburg, Germany). All primary Ab and isotype controls were diluted in PBS pH 7.

**Table 1:** Patient’s clinical information.

**Gender** **Age (Average ± SD; years)**	Male (n = 26) 67 ± 8		Female (n = 24) 66 ± 10	
**Disease Stage**	IIIA (n = 37)	IIIB (n = 1)	IV (n = 12)	
**NSCLC histological subtype**	Adenocarcinoma (n = 24)	Squamous (n = 16)	Large Cell (n = 5)	Pleomorphic (n = 5)

**Table 2:** 

Primary Ab (Clone)	Manufacture	Catalogue	Isotype	Concentration (μg/ml)	Incubation	Isotype control	Antigen retrieval	Detection	Counterstain
CD56 (MRQ-42)	Ventana	760–4596	Rabbit IgG	0.5–1	32 min, 37°C	Cell Signaling, Cat. 3900	Mild CC1	UltraView DAB	Hematoxylin II
CD68 (PG-M1)	Dako	M0876	Mouse IgG3, k	0.06	32 min, 37°C	Biolegend, Cat. 401302	Standard CC1	OptiView DAB	Hematoxylin II
CD8 (C8/144B)	Dako	M7103	Mouse IgG1, k	8	32 min, 37°C	Dako, Cat. X093	Standard CC1	UltraView DAB	Hematoxylin II
FoxP3 (236A/E7)	Abcam	ab20034	Mouse IgG1	10	32 min, 37°C	Dako, Cat. X093	Standard CC1	OptiView DAB	Hematoxylin II
Granzyme B (GrB-7)	Dako	M7235	Mouse IgG2a, k	5.5	60 min, RT	Biolegend, Cat. 400224	Standard CC1	UltraView DAB	Hematoxylin II
PD-L1 (MKP1A07310)	Merck KGaA	Proprietary	Rabbit IgG	0.25	30 min, RT	Cell Signaling, Cat. 3900	TRS high pH	EnVision FLEX/HRP	Hematoxylin EnVision FLEX

Mild CC1: 28 min at 96 °C

Standard CC1: 56 min at 100°C

TRS high pH: 20 min at 95°C

Hematoxylin II: 8 min

Hematoxylin EnVision FLEX: 5 min

### Whole-slide scanning and manual annotation

**Fig 1:**
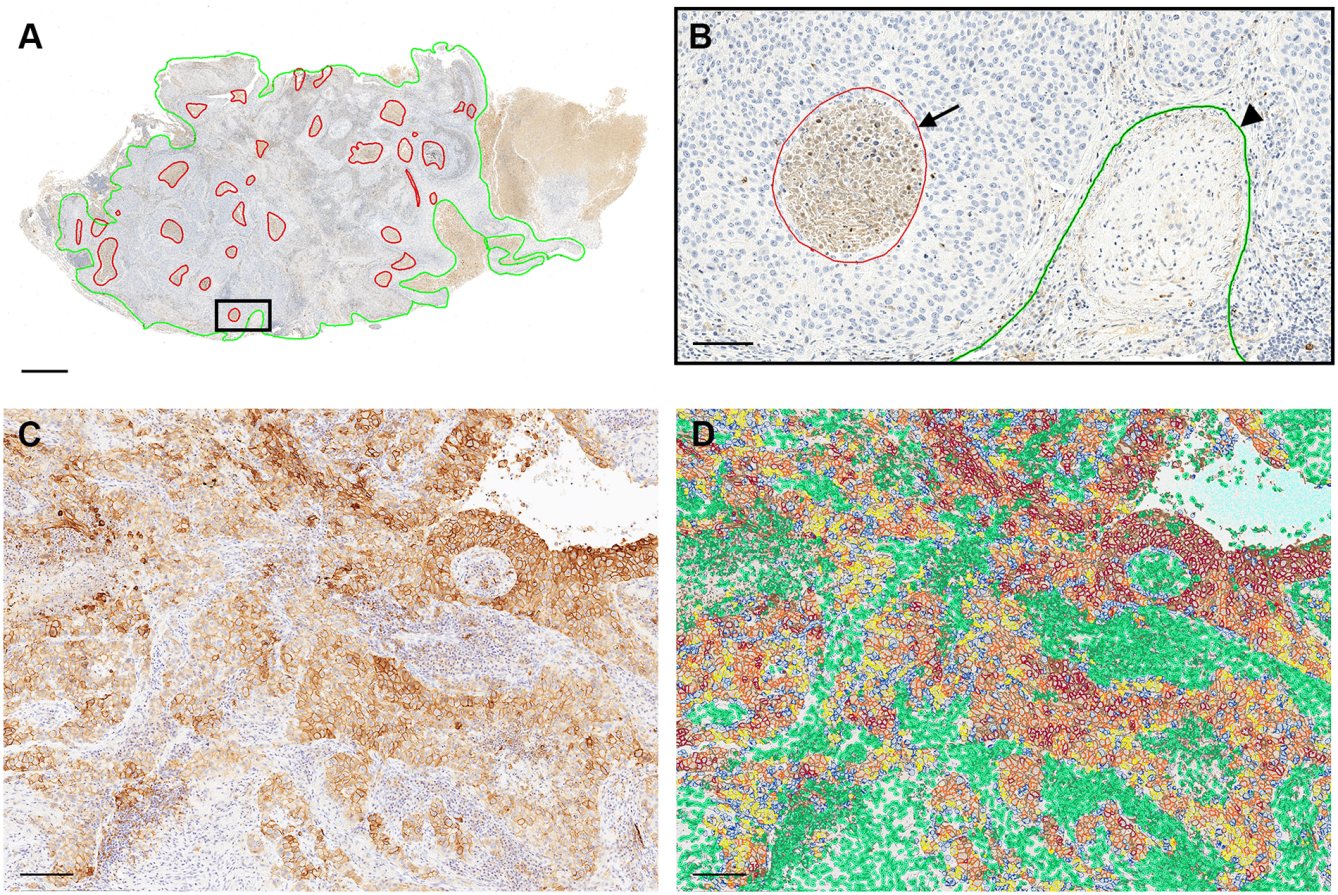
Example of computational tissue analysis (cTA) of NSCLC sections. **A**: Manual inclusion (green line) and exclusion (red line) annotations in squamous cell carcinoma tissue for CD56 IHC. **B**: Enlarged view area from (**A)** showing exclusion annotation around a central necrotic area in tumor-nest (arrow) and a segment of the inclusion annotation around the tumor mass margin (arrowhead). **C**: PD-L1 IHC of a squamous cell carcinoma. **D**: Algorithm markup from (**C)** showing membrane scoring only in cells assigned to the tumor-nest compartment, with 1+ = yellow, 2+ = orange, and 3+ = red. The separated TME compartment cell nuclei are marked in green. Scale bars: 1 mm (**A**); 100 μm (**B**); and 150 μm (**C** and **D**). NSCLC = non-small cell lung carcinoma; IHC = immunohistochemistry; TME = tumor microenvironment.

Slides were digitized using Aperio’s AT Turbo and CS bright-field slide scanning systems (Leica Biosystems Inc., Buffalo Grove, IL) at Flagship Biosciences’ tissue analysis laboratory. A minimum of 4 focal points were set for each slide to allow for optimal scanning quality, with scanning performed at x20. All whole-slide digital images were assessed for scanning artifacts including blurring due to incomplete cleaning, and ‘stitching’ artifacts. Scanning was repeated until all slides reached Flagships’ quality criteria for digital scans. Digital images were viewed using Aperio ImageScope (Leica Biosystems Inc., Buffalo Grove, IL), with manual annotation to include region(s) of interest (ROI). Inclusion annotations were made just outside the invasive margin of the tumor mass when present (Fig 1A and 1B), excluding adjacent pre-existing normal lung and/or pleural tissue and *in-situ* carcinomatous changes. Exclusion annotations were made around staining and tissue artifacts including folds and tears, mounting media artifacts, stain smudges, and other artifacts deemed irreversible in the scanning process. Additional exclusions were areas of necrosis (Fig 1B), entrapped airway tissue (including hyperplastic or metaplastic epithelium), ectopic lymphoid structures, and large intraluminal clusters of macrophages and/or cellular debris. One tissue sample was dropped from the study due to widespread anthracosis.

### Computational tissue analysis (cTA)

Full scan analysis was performed using Flagship Bioscience’s proprietary cTA image analysis software system (Westminster, CO, USA) available for research. The platform identified nuclei in each sample based upon hematoxylin staining, then quantified the intensity of DAB staining for each nucleus-identified cell. To identify positive cells, thresholds for DAB staining were set using biomarker-specific algorithms for CD8, FOXP3, CD56, Granzyme B, CD68, and PD-L1. For PD-L1, multiple thresholds were identified according to a negative, +1, +2, and +3 scoring paradigm contiguous with manual scoring approaches (Fig 1C and 1D). The biomarker-specific algorithms were then further developed on a sample-by-sample basis to assign cells to either the tumor-nest or TME compartments based on morphometric and/or staining qualities (Fig 1D). Instances where compartmental separation was impossible due to similar morphologies of tumor nest and TME cell nuclei, or an insufficient quantity of TME, cell information was studied from both compartments. Data from these cases were included in the “All Cells” category and excluded from the solo tumor-nest and TME compartment-specific analyses. All annotations and image analysis markups were assessed by a pathologist (JPK) to verify performance and accuracy. Stained cell counts and the percentage of positive cells for each tissue sample in every biomarker on a per-compartment basis was performed. Heterogeneity was further analyzed for PD-L1 stained samples using an algorithm-calculated H-Score within the tumor-nest compartment, the TME compartment, and the entire tissue sample respectively. Digital H-scores ranging from 0 to 300 were calculated using the following standard formula: [3 * % cells +3 intensity] + [2 * % cells +2 intensity] + [1 * % cells +1 intensity].

### Pathologist scoring of PD-L1 staining patterns

In addition to the image analysis detailed above, all slide images were reviewed and categorized according to the PD-L1 staining patterns (I-V) noted in tumor-nest and TME compartments, including the tumor-nest-TME interfaces (Table 3). The system used was an adaptation/extension of those previously suggested for cancer classification based on PD-L1 expression by tumor cells and co-localization with TIL [6, 8]. The aim of this categorization was an attempt to differentiate between constitutive versus reactive PD-L1 expression (or mixtures of the two) in order to predict potential responders to PD-1/PD-L1-based immunotherapy [6, 8]. In this study, we assessed PD-L1 expression by all cell types in the TME compartment rather than using TIL counts.

**Table 3:** Classification of NSCLC according to PD-L1 expression patterns in tumor-nest and TME compartments.

Pattern*	PD-L1 expression^†^	Biological relevance
I	TUM+ (diffuse) TME+	Mixed of genetic/epigenetic (constitutive) and adaptive immunity effect (induced)
II	TUM- (patchy) TME+	Only driven by adaptive immunity (induced)
III	TUM- TME-	Immune ignorance
IV	TUM+ TME-	Constitutive only
V	TUM- TME+	Tumor cells might be genetically unable to express PD-L1 and/or functional interferon-γ receptors

* Based on system proposed by Teng *et al*. [8], but with subdivision of category I by Ribas and Hu-Lieskovan [6], and assessment of TME rather than only tumor-infiltrating lymphocytes.

^†^TUM (tumor-nest compartment) and TME (tumor microenvironment compartment). (+) and (-): positive and negative PD-L1 IHC, respectively.

### Statistical analysis

Hierarchical clustering of the data for all biomarkers was performed using the MATLAB bioinformatics toolbox version 2015a (MathWorks, Natick, MA) with Euclidean distance metric and average linkage to generate the hierarchical tree. Data were normalized such that the mean was 0 and the standard deviation was 1 across samples for each marker score. Pearson correlation coefficients were also calculated between marker measurements and a heatmap was generated to visualize the strength of the correlations.

Percentages of cells positive for each biomarker were compared for tumors above and below 5%, 25%, 50%, and 80% cut-off points for PD-L1 immunostaining. These comparisons were made for the tumor-nest, TME, and “All Cells” compartments. Additionally, positive PD-L1 percentages, immune cell percentages, and digital H-scores were compared between the PD-L1 staining pattern categories in the tumor-nest and TME compartments, respectively. Data were plotted as box-and-whisker plots. Boxes show the median and interquartile range (IQR) and whiskers show the range of the data excluding outliers. Outliers are defined as data more than 1.5*IQR beneath the lower quartile or more than 1.5*IQR above the upper quartile. No data points were removed from analysis. *P*-values were calculated using a two-tailed Student’s t-test, assuming unequal sample variances. The Holm method was used to correct for multiple comparisons, with a significance threshold of *P*<0.05 for each subset of analyses.

## Results

### Correlations between immune biomarkers in tumor-nests and TME

**Fig 2:**
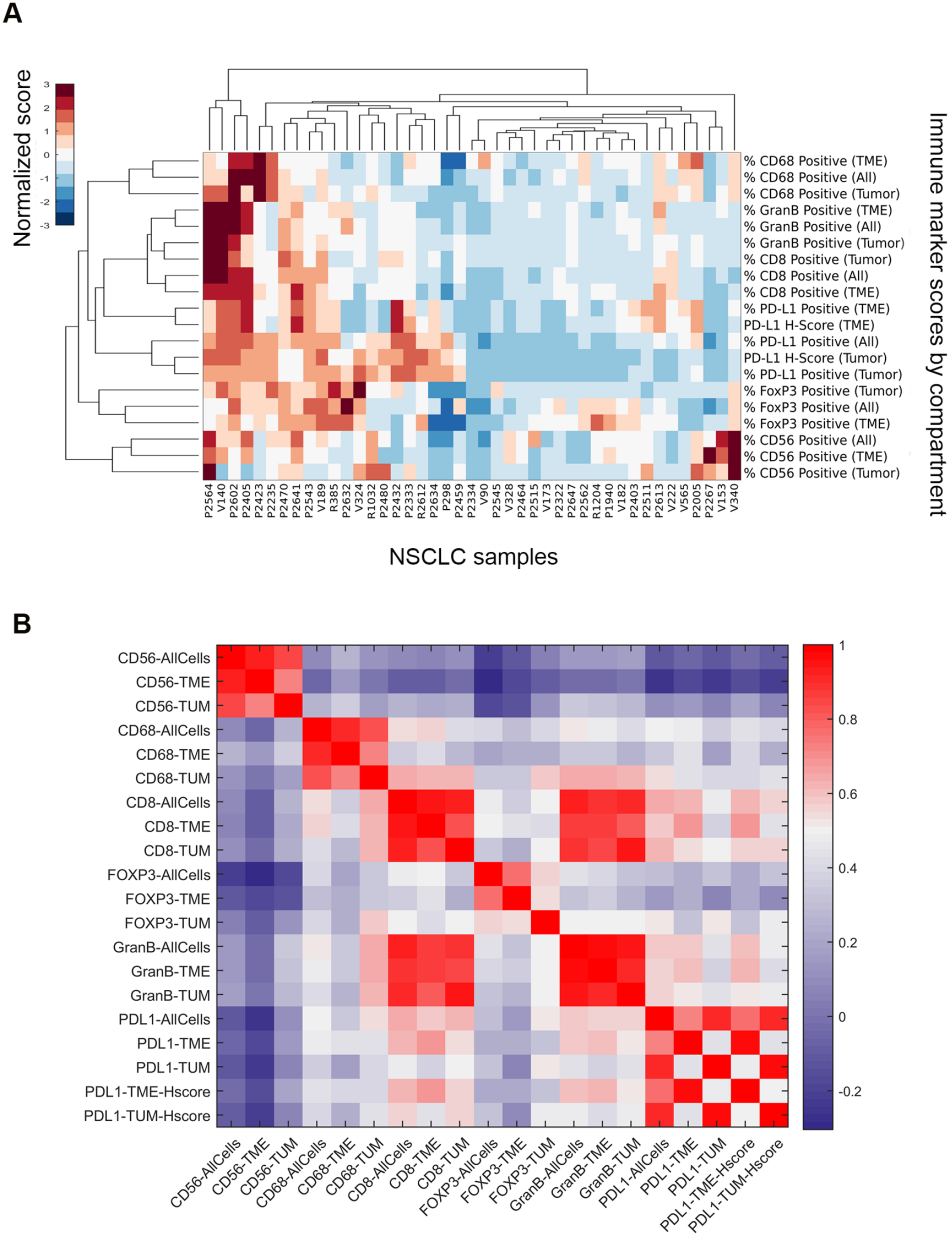
Relationships between biomarkers in our NSCLC cohort. **(A)** Hierarchical clustering heat map of percentages of positive cells for FOXP3, PD-L1, CD8, Granzyme B, and CD68. Each biomarker (row) was normalized before clustering to give a mean of 0 and a standard deviation of 1 across columns (samples). The color scale indicates the relative size of the biomarker score compared to the other samples in the cohort with blue = low, white = neutral, and brown = high. Bracket height indicates the degree of correlation between measurements with shorter brackets representing higher correlations and taller brackets representing smaller correlations. **(B)** Heatmap of Pearson correlation coefficients between percent positive cells measurements across samples for FOXP3, PD-L1, CD8, Granzyme B, and CD68. The color scale ranges from the minimum (blue) and maximum (red) correlation coefficient observed in this analysis. Therefore red represents strong positive correlations, white represents moderate correlations, and blue represents weak correlations. No strong negative correlations were observed.

Multiple biomarkers were analyzed in the tumor-nest, TME, and All Cells compartments. This allowed us to accurately assess the relative proportions of effector and regulatory cells in the typically heterogeneous NSCLC tissue microenvironment, and their possible relationships. In a small number of cases (n = 7), it was not possible to separate tumor-nest and TME compartments digitally as the latter was minimal; All Cells scores only were analyzed for those slides. In the group of cases where a separation between tumor-nest and microenvironment was feasible (n = 43), scores derived from a single marker showed a greater correlation across compartments than scores derived from different markers in the same compartment (Fig 2A). For example, percentages of CD68-positive cells in the tumor-nest and TME compartments had a Pearson correlation coefficient (ρ) of 0.73, which fell to 0.42 when comparing CD68 and CD8 in the TME alone. However, PD-L1 data showed a lower correlation between the tumor-nest and TME compartments (ρ = 0.43) than the other markers (Fig 2B). CD56 showed the least correlation with other markers, while CD8 and Granzyme B (both expressed by cytotoxic T-cells) showed the greatest association with each other (All Cells ρ = 0.93, *P* < 0.0001; Fig 2B). Of the biomarkers beside PD-L1, CD8- and CD68-positive cells had the highest mean percentages in both tumor-nest (10.7%; 8.3%) and TME compartments (11.1%; 13.2%) (Table 4). Finally, we could not identify significant clustering based on the four NSCLC subtypes represented in our sample set (adenocarcinoma, squamous cell carcinoma, large cell carcinoma, and pleomorphic carcinoma).

**Table 4:** Average percent positive cells for multiple immune biomarkers in NSCLC tissue compartments.

Biomarker	All Cells* (n = 50)	Tumor-nests (n = 43)	Tumor microenvironment (TME) (n = 43)
PD-L1	32.90	35.60	31.40
CD8	8.36	6.02	11.11
Granzyme B	3.17	2.05	4.41
FOXP3	4.42	1.81	7.16
CD68	8.61	4.14	13.43
CD56	0.63	0.22	1.07

*“All Cells” is the combined data for tumor-nest and TME

### Distribution of immune biomarkers relative to frequently used PD-L1 cut-off points

**Fig 3:**
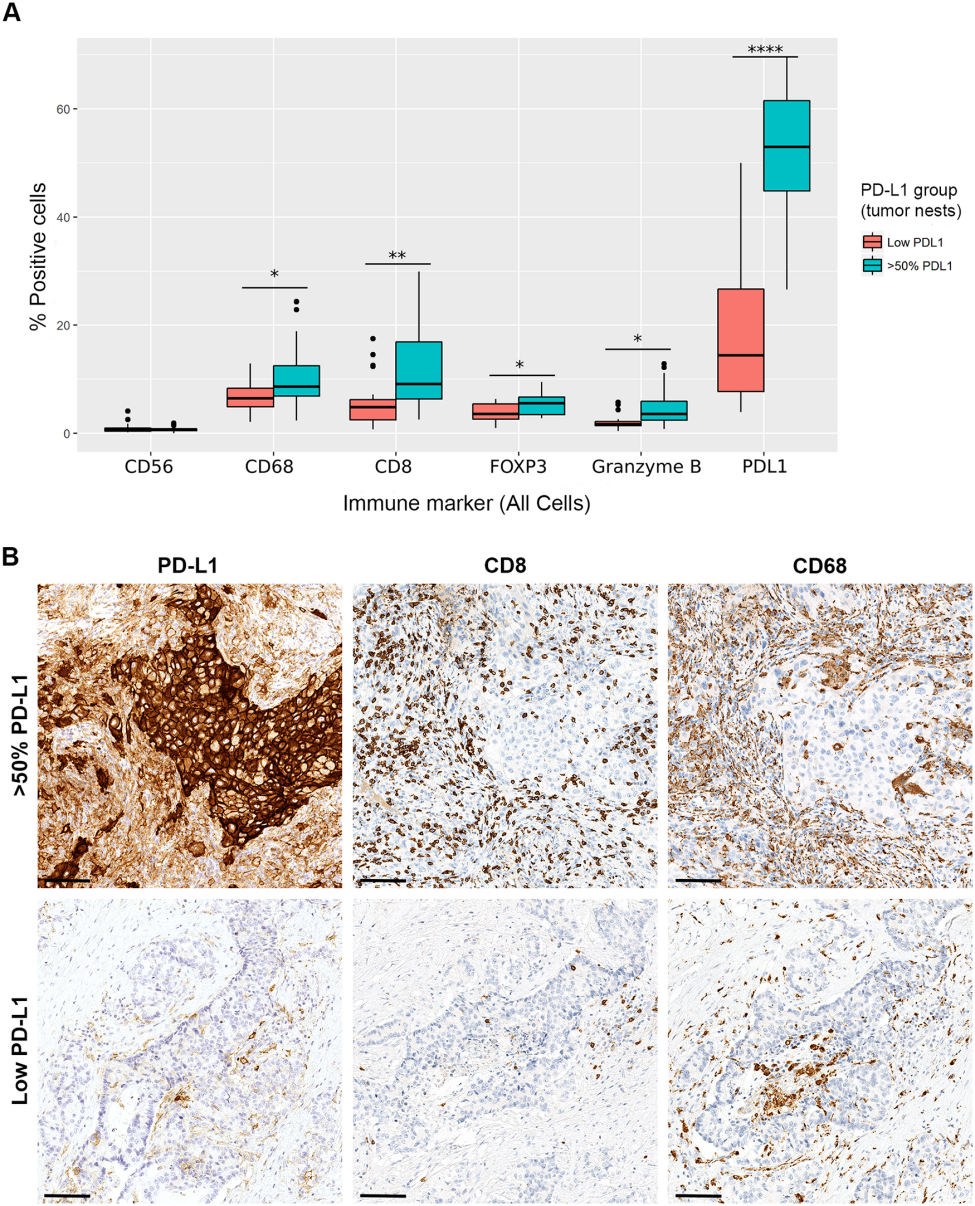
Immune markers in NSCLC relative to a 50% cut-off point for PD-L1 staining. **A**: Percentage of positive cells for each biomarker in “All Cells” (tumor nest and TME compartments combined) calculated by using Computational Tissue Analysis (cTA^™^). NSCLC with >50% PD-L1-positive tumor cells were compared with those with lower expression. Statistical differences were calculated using two-tailed Student’s t-tests, assuming unequal sample variances (*P ≤ 0.05, **P ≤ 0.01, ***P ≤ 0.001 and ****P ≤ 0.0001). Percentages of cells positive for CD68, CD8, FoxP3, and Granzyme B were significantly higher in the >50% PD-L1 group. **B**: Representative IHC images of PD-L1-, CD8-, and CD68-positive cells (the most numerous biomarkers) in an NSCLC (solid carcinoma) case with >50% PD-L1 expression in the tumor nest compartment, and an NSCLC (adenocarcinoma with mixed subtypes) case with lower PD-L1 expression. Scale bars = 100 μm. NSCLC = non-small cell lung carcinoma; IHC = immunohistochemistry; TME = tumor microenvironment.

We compared the distributions of our immune markers relative to PD-L1 positive staining cut-offs (5, 25, 50, and 80%) currently used in Merck KGaA PD-L1 clinical development programs. We studied cut-off points in the tumor nest, TME, and All Cells compartments, with a full analysis of the immune markers in each compartment. A 50% PD-L1 TME cut-off put only 14% of samples in the high PD-L1 group, increasing to 100% at a 5% PD-L1 TME cut-off. Whereas, data from tumor-nest cut-offs permitted a more balanced sample size distribution. For example, using the 25% and 50% PD-L1 tumor-nest cut-offs, approximately half of the samples were in the high PD-L1 group (53% and 44%, respectively). A plot of 50% PD-L1 tumor-nest cut-off (most frequently scored by pathologists) for overall immune cell expression (All Cells) provides a summary of the global changes in cellular distribution for the PD-L1 high/low groups (Fig 3A). Higher percentages (*P* < 0.05) of stained cells for CD68, CD8, FOXP3, and Granzyme B were detected in the high PD-L1 group when compared vs the low PD-L1 group (Fig 3A). Results were similar when comparing immune marker distribution in the tumor-nest, TME, and All Cells compartments using 25% and 50% PD-L1 tumor-nest cut-off points (non-shown data). Additionally, we found no significant differences in Granzyme B/CD8 ratio for the 50% cut-off (*P* = 0.14), but a higher FOXP3/CD8 ratio beneath the 50% threshold (*P* = 0.03). When applying a 5% PD-L1 tumor-nest cut-off, 70% of samples segregated to the high PD-L1 group, with CD8, Granzyme B, and CD68 significantly higher in those samples (*P* < 0.05). Statistical comparisons using an 80% PD-L1 tumor-nest cut-off value were impossible given that only one sample segregated to the high PD-L1 group.

### Classification of PD-L1 staining patterns and relation with cut-Off points

**Fig 4:**
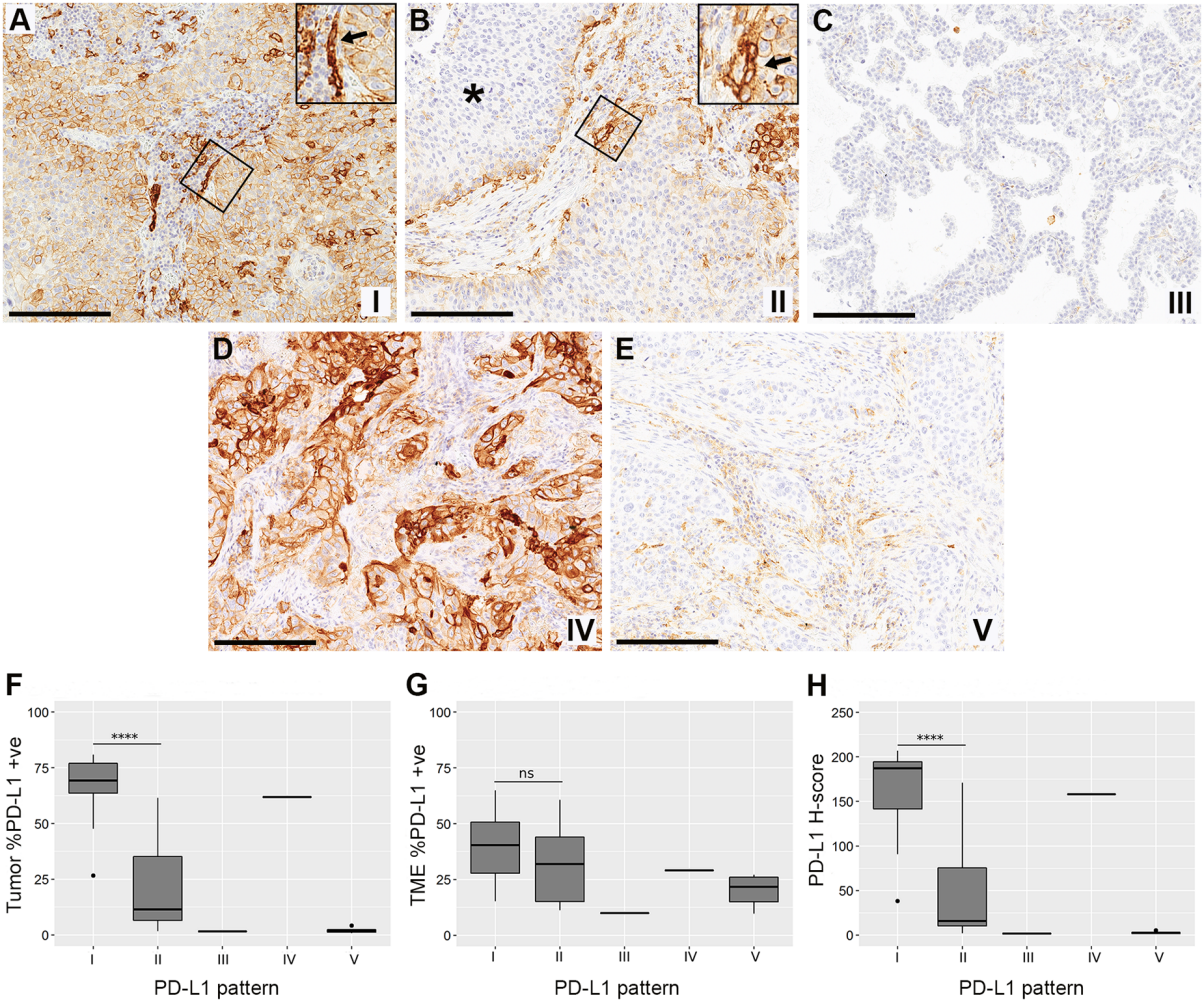
Examples of PD-L1 staining patterns observed and categorized in this NSCLC series. **A**: Pattern I—Constitutive mixed with induced. Diffuse expression of PD-L1 (IHC) on tumor cell membranes of a squamous cell carcinoma, including central regions of trabeculae. Prominent labeling of cells in the TME compartment at the tumor-nest-TME interface suggesting presence of an immunological synapse (inset arrow). **B**: Pattern II—Induced only. Patchy expression of PD-L1 in a squamous cell carcinoma at the tumor-nest-TME interface (inset arrow). Minimal to no PD-L1 expression in the trabeculae (asterisk) if compared with (**A**). **C**: Pattern III—Immune ignorance. No to minimal PD-L1 expression in both tumor and TME compartments in an adenocarcinoma. **D**: Pattern IV—Constitutive only. Diffuse expression of PD-L1 by tumor-nests in an adenocarcinoma with minimal TME staining. **E**: Pattern V—TME expression only. No to minimal PD-L1 expression in tumor cells of a squamous cell carcinoma, with widespread staining in the TME compartment. **F**: Percentages of PD-L1-positive tumor cells in all staining pattern categories (I-V). **G**: Percentages of TME PD-L1-positive cells in all staining pattern categories (I-V). **H**: Digital H-scores from tumor-nests in all staining pattern categories (I-V). Significant differences between groups I and II were calculated using two-tailed Student’s t-tests, assuming unequal sample variances (*P ≤ 0.05, **P ≤ 0.01, ***P ≤ 0.001 and ****P ≤ 0.0001). Scale bars: 200 μm (**A-E**). NSCLC = non-small cell lung carcinoma; IHC = immunohistochemistry; TME = tumor microenvironment.

The staining patterns for PD-L1 (Table 3) were classified for cases with tumor-nest and TME compartment data (n = 43) to determine whether any NSCLC case with evidence of reactive membrane expression would be falsely excluded at certain cut-off points, while some NSCLC with only constitutive tumor expression might be inappropriately included. Most of the tumors in this sample set were classified as having pattern I (n = 16) or II (n = 16); both showing PD-L1 expression in tumor cells at the tumor-nest-TME interface that suggested contribution of a reactive process (Fig 4A and 4B).

In NSCLC classified as pattern I, the tumor cells showed variable percentages of PD-L1 membrane labeling (1+, 2+, and 3+ scores) and positive staining in TME cells (Fig 4A). Heavier PD-L1 staining of tumor cells adjacent to positive TME cells were observed by the pathologist in some fields, which varied within and between sections. In tumors classified as pattern II, the variable staining intensity was largely limited to patches that were often located at the tumor-nest-TME interface (Fig 4B). PD-L1 positive cells in the TME for pattern I and II included morphological cellular components compatible with lymphocytes, macrophages, fibroblasts, and endothelial cells. Looking at the other immune biomarkers, CD8 and to lesser extent CD68, most often showed pronounced infiltration on both sides of the tumor-nest-TME interface, as opposed to the other immune markers that tended to be more diffusely arranged in both compartments. Only one tumor showed predominantly negative staining for PD-L1 in both compartments (tumor-nest and TME), consistent with pattern III (Fig 4C). Pattern IV (diffuse and intense staining of tumor cell membranes with minimal to no TME expression) was also only noted in one case (Fig 4D). Finally, there were a number of NSCLC cases (n = 9) with PD-L1 staining in the TME of variable intensity and minimal to no tumor cell staining consistent with pattern V (Fig 4E).

Comparison of tumor groups with patterns I and II revealed a lower percentage of PD-L1 positive cells in the tumor-nests from the group showing pattern II staining (*P* < 0.00001). Importantly, most of those pattern II samples fell beneath the cut-off points when applying 50% and 25% PD-L1 tumor-nest thresholds (Fig 4F). However, comparable percentages of PD-L1-positive TME cells were noted for each of these patterns (Fig 4G). A similar separation of these two groups could be achieved by using a tumor-nest digital H-score, with pattern I and II groups having significantly different averages of 160 and 41, respectively (Fig 4H). In terms of other immune biomarker cellular percentages (and their digital H-scores), there were no significant differences between pattern I and II groups for TME or All Cells analyses. However, the tumor nest compartment for pattern I tumors showed significantly higher percent of FOXP3-positive cells (*P* = 0.002; data not shown).

## Discussion

Using procured NSCLC tissues from naïve-treated patients with advanced disease, we adopted the cTA platform to identify and quantify multiple immune biomarkers in tumor-nest and TME compartments in whole sections. Pivotal to this analysis was the usage of the Merck KGaA anti-PD-L1 clone MKP1A07310, which is the analytical Ab for the evaluation of Avelumab [5], with same specificity and higher sensitivity than Dako anti-PD-L1 clone 22C3 as shown in a NSCLC PD-L1 IHC assay comparison study [14]. We demonstrated that all our immune biomarkers, except for PD-L1, had a strong correlation across tumor-nest and TME compartments, with CD8 and Granzyme B being the TIL markers with strongest association. PD-L1 tumor-nest cut-off points, but not TME-related ones, allowed a balanced sample size distribution that showed similar results at 25 and 50% in terms of TIL-related and non-related immune biomarkers, clustering higher levels of these immune biomarkers with higher levels of PD-L1 expression. This is consistent with the hypothesis that higher PD-L1 tumor-nest expression would be expected in the context of an adaptive immune response to tumor antigens. However, we propose that a simple quantification of PD-L1 immunostaining levels is not enough to select patients for PD-1/PD-L1 immunotherapy [6], because cut-off points do not allow discrimination between constitutive and reactive expression patterns. Our data show that most cases with a patchy PD-L1 reactive pattern II fell below both 25 and 50% cut-off points, indicating that they would be excluded from PD-1/PD-L1 immunotherapy if those cut-off criteria were used for patient selection. Also, the inclusion of cases with high PD-L1 tumor expression related to a constitutive expression either alone (pattern IV) or predominant (pattern I) may not necessarily favor therapeutic outcome. In conclusion, we propose that current scoring systems based simply on amount of staining should be complemented by algorithms that discriminate between constitutive and reactive PD-L1 staining patterns, being the last one the appropriate target for PD-1/PD-L1 checkpoint inhibition.

We have demonstrated enrichment of effector and regulatory immune cell types in groups with higher PD-L1 expression as defined by IHC thresholds of over 25 and 50%. This included CD8 and Granzyme B positive cells, with the latter being a marker that highlights functional effector T-cells [15]. The surprisingly low percentage of Granzyme B^+^ cells versus total CD8^+^ cells in our study might be explained by a large proportion of exhausted CD8 T cells [9, 16]. The Granzyme B^+^/CD8^+^ ratio did not differ significantly across the 25% and 50% thresholds, but might not be expected in our particular cohort of patients with advanced disease. Increased percentages of FOXP3^+^ cells with higher PD-L1 expression also suggested a tumor cell modulation of the adaptive immune response given that PD-L1/PD-1 interactions promote T-cells differentiation into regulatory cells [17]. The balance between FOXP3^+^ and CD8^+^ T-cells is another clinically linked parameter, as low-FOXP3/ high-CD8^+^ ratios have been linked with positive survival effects across multiple cancer types including NSCLC [18–19]. In the current study, the FOXP3^+^/CD8^+^ ratio was lower in NSCLC above the 50% PD-L1 cut-off point, but this result does not necessarily indicate a favorable TME for an adaptive anti-tumoral immune response due to the likelihood that most of those TIL are exhausted. Overall, these results support using PD-L1 immunostaining cut-off points to select patients with higher chances of responding to PD-1/PD-L1 checkpoint inhibition, as they are consistent with previous positive correlations of PD-L1 expression, numbers of TIL, and longer overall survival in NSCLC and other solid tumors [15, 20–21, 22–23]. However, our further analysis of PD-L1 staining patterns in tumor-nests and TME suggested the need to take into account this additional aspect.

The categorization of tumor PD-L1 expression patterns related to TIL distribution has been discussed previously, suggesting clear guidelines for checkpoint inhibition immunotherapy [6, 8]. This approach may simplify PD-L1 scoring paradigms, but detracts from complex immunologic read-outs reflecting the contribution from various combinations of immune biomarkers. In fact, tumor-associated macrophages, fibroblasts, dendritic cells, and even endothelial cells can modulate immune responses via PD-L1 expression [16, 24–30]. PD-L1 may also be expressed on T-cells themselves [31]. Cells with morphologies consistent with all these cell types were found to express variable PD-L1 labeling intensities in our tissue sections classified as pattern I, II and V. Moreover, these PD-L1 expressing cells have been previously shown to be therapeutically important, as positive responses to Atezolizumab were enriched in NSCLC when PD-L1 was expressed by infiltrating immune cells [31]. In fact, absence of PD-L1 expression in the immune infiltrate was categorized as a “non-functional immune response” [31]. PD-L1 expression in TME immune and non-immune cells could be an over-arching biomarker for the anti-tumor response, allowing selection of appropriate patients for checkpoint inhibitor immunotherapy. However, it is unknown which are the characteristics of the PD-L1 staining required in the TME (pattern I, II and V) for a successful therapeutic intervention. In our study, the TME expression of PD-L1 alone did not facilitate separation of patients using cut-off points, but it’s relationship to tumor cell staining in the context of our PD-L1 pattern classification system (I-V) suggested why simple cut-off points may exclude therapy-responsive patients, while including some who are unlikely to respond.

The analysis of PD-L1 staining patterns of tumor cells was performed to titrate the presence of morphological structures where PD-L1/PD-1 interactions should occur like an ‘immunologic synapse’. This is the classic TCR/antigen presentation interface where T-cell immune activation and inhibitory signals interplay [32]. In our tissue context, localization of TIL at the tumor-nest-TME interface and among other PD-L1-expressing cell types within the TME compartment (captured in our pattern I, II and V) provide ample opportunities for PD-L1/PD-1 interactions to attenuate or paralyze any adaptive immune response. This physical interaction was most clearly seen in our staining pattern II, which had a prominent and patchy tumor-nest-TME interface (Fig 4B). In contrast, where PD-L1 expression by the tumor cells is diffuse, often intense, and not only localized to the interface (patterns I or III), it is logical to assume that this expression reflects tumoral-intrinsic events related to genetic mutation or epigenetic disturbance [6]. Such tumors will tend to be given high scores that are out of proportion to the numbers of infiltrating immune cells [33], although many of such cases in our study did also contain interface components (pattern I). We hypothesized that NSCLC tissues with interface-only staining (pattern II) as well as the cases showing mixed interface and diffuse staining (pattern I) contain inducible PD-L1/PD-1 interactions potentially adequate for therapeutic blocking. Of note, current NSCLC PD-L1 scoring methods do not allow us to identify patterns I and II [3], as traditional cut-off point fail to recognize these essential interactions at the interface. Moreover, we have shown that most of the pattern II tumors fell below a 25% and 50% cut-off points, which are commonly used in current scoring methods [3]. Also, these methods will not assess if a sufficient extent and/or magnitude of adaptive immunity is present to select patients who could benefit from PD-1/PD-L1 therapeutic intervention. In fact, some pattern II tumors may contain a more extensive and targetable adaptive immune response than those included in the pattern I category. Setting a lower tumor cell cut-off point (e.g. 1% or 5%) risks including pattern II without sufficient immunological synapse interfaces, while still including potentially recalcitrant pattern I or III tumors. Then, current scoring algorithms based on amount of staining should be complemented with qualitative algorithms that allow the discrimination between constitutive and relevant reactive PD-L1 staining patterns.

In conclusion, using our cTA analysis of PD-L1 immunostaining relative to immune biomarkers that might be involved in NSCLC immune surveillance during this late disease stage, we demonstrated that groups with high levels of PD-L1 defined by cut-off points of 25 and 50% enriched also for higher amounts of FOxP3, CD8, Granzyme B, and CD68 positive cells in tumor-nest and TME. This association between high levels of PD-L1 and increased effector and suppressor adaptive as well as augmented innate immune tumor infiltrates may reflects escape from a potential host anti-tumoral immune response. Furthermore, high amounts of PD-L1 in NSCLC defined by these or other cut-off points may not accurately predict if anti-PD-1/PD-L1 therapy will successfully unleash a host anti-tumoral immune response. Selection of a specifically reactive PD-L1 staining pattern within regions of the tumor architecture where immunological synapses are formed is likely to be required for more accurate determination of which patients will benefit from this therapeutic intervention. We anticipate that the current data may trigger a new way to analyze clinical trial specimens, because discrimination between constitutive and reactive PD-L1 staining patterns should improve the precision of immunotherapy patient selection.

## References

[1] Dela Cruz CS, Tanoue LT, Matthay RA. Lung cancer: epidemiology, etiology and prevention. Clin Chest Med 2011; 32:605–644. doi: 10.1016/j.ccm.2011.09.001 22054876 10.1016/j.ccm.2011.09.001 PMC3864624

[2] Schiller JH, Harrington D, Belani CP, Langer C, Sandler A, Krook J, et al Eastern Oncology Cooperative Group. Comparison of four chemotherapy regimens for advanced non-small-cell lung cancer. N Engl J Med 2002; 346:92–98. doi: 10.1056/NEJMoa011954 11784875 10.1056/NEJMoa011954

[3] The I ASLC atlas of PD-L1 immunohistochemistry testing in lung cancer. https://www.iaslc.org/publications/iaslc-atlas-pd-l1-testing-lung-cancer.

[4] Grigg C, Rizvi NA. PD-L1 biomarker testing for non-small cell lung cancer: truth or fiction? J Immunother Cancer 2016; 4:48 doi: 10.1186/s40425-016-0153-x 27532023 10.1186/s40425-016-0153-x PMC4986262

[5] Gulley JL, Rajan A, Spigel DR, Iannotti N, Chandler J, Wong DJL, et al Avelumab for patients with previously treated metastatic or recurrent non-small-cell lung cancer (JAVELIN Solid Tumor): dose-expansion cohort of a multicentre, open-label, phase 1b trial. Lancet Oncology 2017; 18:599–610. doi: 10.1016/S1470-2045(17)30240-1 28373005 10.1016/S1470-2045(17)30240-1 PMC5522719

[6] Ribas A, Hu-Lieskovan S. What does PD-L1 positive or negative mean? J Exp Med 2016; 213:2835–2840. doi: 10.1084/jem.20161462 27903604 10.1084/jem.20161462 PMC5154949

[7] Ilie M, Hofman V, Dietel M, Soria JC, Hofman P. Assessment of the PD-L1 status by immunohistochemistry: challenges and perspectives for therapeutic strategies in lung cancer patients. Virchows Arch 2016; 468:511–525. doi: 10.1007/s00428-016-1910-4 26915032 10.1007/s00428-016-1910-4

[8] Teng MWL, Ngiow SF, Ribas A, Smyth MJ. Classifying cancers based on T cell infiltration and PD-L1. Cancer Res 2015; 75:2139–2145. doi: 10.1158/0008-5472.CAN-15-0255 25977340 10.1158/0008-5472.CAN-15-0255 PMC4452411

[9] Prado-Garcia H, Romero-Garcia S, Puerto-Aquino A, Rumbo-Nava U. The PD-L1/PD-1 pathway promotes dysfunction, but not “exhaustion”, in tumor-responding T cells from pleural effusions in lung cancer patients. Cancer Immunol Immunother 2017; 66:765–776. doi: 10.1007/s00262-017-1979-x 28289860 10.1007/s00262-017-1979-x PMC11028517

[10] Taube JM, Anders RA, Young GD, Xu H, Sharma H, McMiller TL, et al Colocalization of inflammatory responses with B7-H1 expression in human melanocytic lesions supports an adaptive resistance mechanism of immune escape. Sci Transl Med 2012; 4:127ra37 doi: 10.1126/scitranslmed.3003689 22461641 10.1126/scitranslmed.3003689 PMC3568523

[11] Chen DS, Mellman I. Elements of cancer immunity and the cancer-immune set point. Nature 2017; 541:321–330. doi: 10.1038/nature21349 28102259 10.1038/nature21349

[12] Spranger S. Mechanisms of tumor escape in the context of the T-cell-inflamed and the non-T-cell-inflamed tumor microenvironment. Int Immunol 2016; 28:383–391. doi: 10.1093/intimm/dxw014 26989092 10.1093/intimm/dxw014 PMC4986232

[13] Parra ER, Behrens C, Rodriguez-Canales J, Lin H, Mino B, Blando J, et al Image Analysis-based Assessment of PD-L1 and Tumor-Associated Immune Cells Density Supports Distinct Intratumoral Microenvironment Groups in Non-small Cell Lung Carcinoma Patients. Clin Cancer Res 2016; 22:6278–6289. doi: 10.1158/1078-0432.CCR-15-2443 27252415 10.1158/1078-0432.CCR-15-2443 PMC5558040

[14] Feng Z, Schlichting M, Helwig C, Chand VK, Gelb A, Jin H. Comparative study of two PD-L1 expression assays in patients with non-small cell lung cancer (NSCLC). J Clin Oncol 2017; 35:e20581.

[15] Nowacki TM, Kuerten S, Zhang W, Shive CL, Kreher CR, Boehm BO, et al Granzyme B production distinguishes recently activated CD8^+^ memory cells from resting memory cells. Cell Immunol 2007; 247:36–48. doi: 10.1016/j.cellimm.2007.07.004 17825804 10.1016/j.cellimm.2007.07.004 PMC2134935

[16] Jiang Y, Li Y, Zhu B. T-cell exhaustion in the tumor microenvironment. Cell Death Dis 2015; 6:e1792 doi: 10.1038/cddis.2015.162 26086965 10.1038/cddis.2015.162 PMC4669840

[17] Barber DL, Wherry EJ, Masopust D, Zhu B, Allison JP, Sharpe AH, et al Restoring function in exhausted CD8 T cells during chronic viral infection. Nature 2006; 439:682–687. doi: 10.1038/nature04444 16382236 10.1038/nature04444

[18] Gooden MJ, de Bock GH, Leffers N, Daemen T, Nijman HW. The prognostic influence of tumor-infiltrating lymphocytes in cancer: a systematic review with meta-analysis. Br J Cancer 2011; 105:93–103. doi: 10.1038/bjc.2011.189 21629244 10.1038/bjc.2011.189 PMC3137407

[19] Teng F, Meng X, Wang X, Yuan J, Liu S, Mu D, et al Expressions of CD8+TILs, PD-L1 and Foxp3+ TILs in stage I NSCLC guiding adjuvant chemotherapy decisions. Oncotarget 2016; 7:64318–64329. doi: 10.18632/oncotarget.11793 27602763 10.18632/oncotarget.11793 PMC5325445

[20] Schalper KA, Brown J, Carvajal-Hausdorf D, McLaughlin J, Velcheti V, Syrigos KN, et al Objective measurement and clinical significance of TILs in non-small cell lung cancer. J Natl Cancer Inst 2015; 107:dju435. doi: 10.1093/jnci/dju435 25650315 10.1093/jnci/dju435 PMC4565530

[21] Velcheti V, Schalper KA, Carvajel DE, Anagnostou K, Syrigos KN, Sznol M, et al Programmed death ligand-1 expression on non-small cell lung cancer. Lab Invest 2014; 94:107–116. doi: 10.1038/labinvest.2013.130 24217091 10.1038/labinvest.2013.130 PMC6125250

[22] Droeser RA, Hirt C, Viehl CT, Frey DM, Nebiker C, Huber X, et al Clinical impact of programmed cell death ligand 1 expression in colorectal cancer. Eur J Cancer 2013; 49:2233–2242. doi: 10.1016/j.ejca.2013.02.015 23478000 10.1016/j.ejca.2013.02.015

[23] Lipson EJ, Vincent JG, Loyo M, Kagohara LT, Luber BS, Wang H, et al PD-L1 expression in the Merkel cell carcinoma microenvironment: association with inflammation, Merkel cell polyomavirus, and overall survival. Cancer Immunol Res 2013; 1:54–63. doi: 10.1158/2326-6066.CIR-13-0034 24416729 10.1158/2326-6066.CIR-13-0034 PMC3885978

[24] Zhang X, Mosser DM. Macrophage activation by endogenous danger signals. J Pathol 2008; 214:161–176. doi: 10.1002/path.2284 18161744 10.1002/path.2284 PMC2724989

[25] Lee Y-J, Moon Y-H, Hyung KE, Yoo J-S, Lee MJ, Lee IH, et al Macrophage PD-L1 strikes back: PD-1/PD-L1 interaction drives macrophages towards regulatory subsets. Adv Biosci Biotechnol 2013; 4:19–29.

[26] Ferenbach D, Hughes J. Macrophages and dendritic cells: what is the difference? Kidney Int 2008; 74:5–7. 18560360 10.1038/ki.2008.189

[27] Mu CY, Huang JA, Chen Y, Chen C, Zhang XG. High expression of PD-L1 in lung cancer may contribute to poor prognosis and tumor cells immune escape through suppressing tumor infiltrating dendritic cells maturation. Med Oncol 2011; 28:682–688. doi: 10.1007/s12032-010-9515-2 20373055 10.1007/s12032-010-9515-2

[28] Nazareth MR, Broderick L, Simpson-Abelson MR, Kelleher RJ, Yokota SJ, Bankert RB. Characterization of human lung tumor-associated fibroblasts and their ability to modulate the activation of tumor-associated T-cells. J Immunol 2007; 178:5552–5562. 17442937 10.4049/jimmunol.178.9.5552

[29] He H, Qi L, Xiao Y, Hou Y. Tumor-associated fibroblasts promote PD-L1 expression in lung cancer cells. Zhongguo Fei Ai Za Zhi 2017; 20;293–297. doi: 10.3779/j.issn.1009-3419.2017.05.01 28532535 10.3779/j.issn.1009-3419.2017.05.01 PMC5973068

[30] Rodig N, Ryan T, Allen JA, Pang H, Grabie N, Chernova T, et al Endothelial expression of PD-L1 and PD-L2 down-regulates T cell activation and cytolysis. Eur J Immunol 2003; 33:3117–3126. doi: 10.1002/eji.200324270 14579280 10.1002/eji.200324270

[31] Herbst RS, Soria JC, Kowanetz M, Fine GD, Hamid O, Gordon MS, et al Predictive correlates of response to the anti-PD-L1 antibody MPDL3280A in cancer patients. Nature 2014; 515:563–567. doi: 10.1038/nature14011 25428504 10.1038/nature14011 PMC4836193

[32] Dustin ML. The immunological synapse. Cancer Immunol Res 2014; 2:1023–1033. doi: 10.1158/2326-6066.CIR-14-0161 25367977 10.1158/2326-6066.CIR-14-0161 PMC4692051

[33] Taube JM, Klein A, Brahmer JR, Xu H, Pan X, Kim JH, et al Association of PD-1, PD-1 ligands, and other features of the tumor immune microenvironment with response to anti-PD-1 therapy. Clin Cancer Res 2014; 20:5064–5074. doi: 10.1158/1078-0432.CCR-13-3271 24714771 10.1158/1078-0432.CCR-13-3271 PMC4185001

